# Who gets a mammogram amongst European women aged 50-69 years?

**DOI:** 10.1186/2191-1991-2-6

**Published:** 2012-04-05

**Authors:** Ansgar wuebker

**Affiliations:** 1

**Keywords:** Mammogram, Physician quality, Life expectancy, Instrumental variables

## Abstract

On the basis of the Survey of Health, Ageing, and Retirement (SHARE), we analyse the determinants of who engages in mammography screening focusing on European women aged 50-69 years. A special emphasis is put on the measurement error of subjective life expectancy and on the measurement and impact of physician quality. Our main findings are that physician quality, better education, having a partner, younger age and better health are associated with higher rates of receipt. The impact of subjective life expectancy on screening decision substantially increases after taking measurement error into account.

**JEL Classification:**

C 36, I 11, I 18

## Introduction

Breast cancer is the most common cause of cancer deaths among women in the member states of the European Union [1]. According to estimates of incidence and mortality by the International Agency for Research on Cancer (IARC), there were 331,000 new cases and 90,000 deaths due to breast cancer in the EU in 2006 [1]. Breast cancer accounts for almost one out of three new cancer cases and one out of six (17%) cancer deaths. One in nine women gets breast cancer at some point in her life and one in thirty perishes as a consequence of the disease [2]. Due to demographic trends, significantly more women per capita will be confronted with this disease in the future [3].^A ^Moreover breast cancer is associated with high costs for national health care accounting to about 0.5-0.6% of the total health care expenditure of developed countries [2].

Breast cancer takes years to develop. At the onset of the disease, most breast cancers cause no symptoms. As long as cancer has not metastasized, i.e. that has not moved to the lymph system or to other organs of the body, patients have a five-year survival rate of 96%. If the cancer has spread to the nearby lymph nodes, the rate drops down to 81%. Women whose breast cancer has metastasized to other organs of the body have a five-year survival rate of 26% [4].

A mammogram screening is the best tool available for detecting breast cancer in the early stage, i.e. before symptoms appear. Mammography can detect a breast lump before it can be palpated; it can save live by detecting breast cancer in the earliest stage. For women aged 50- 69 years, mammography has been shown to lower the risk of dying from breast cancer by 35% [4]. In addition it has shown to be highly cost-effective for women in this age group [5]. In light of the evidence available, the International Agency for Research on Cancer expert working group (IARC Working Group, [6]) advises that mammography screening should be offered as a public health policy directed to women aged 50-69 every two years in order to reduce the risk of death from breast cancer. EU guidelines [7] promote a target screening rate of at least 75% of eligible women in European countries. Even though mammography is officially recommended both on the national and European level, screening rates in most European countries remain far from 100%. ^B ^For example, in the Slovak Republic only around 20% of women aged 50-69 years are screened annually [2]. Correspondingly, increasing mammography for women aged 50-69 years is an important public health goal in Europe [1].

There exists a considerable amount of empirical and theoretical research in health economics on the predictors of screening and preventive behaviour. Theoretical economic models include those of Grossman [8], Cropper [9], Giuffrida and Gravelle [10], Byrne and Thompson [11], Howard [12] or Fang and Wang [4]. Jepson et al. [13] and Schueler et al. [14] provide good reviews of the empirical literature on determinants of mammography screening uptake and recommendations for increasing uptake. Although the literature on factors associated with mammography screening is abundant, the reasons for underparticipation remain unclear, because empirical results are inconclusive and still incomplete. Identifying the reasons behind lower screening rates is of high importance, since screening is a crucial first step in the process of early detection and treatment. Once the disease is detected, medical providers and the health care system have a major influence in what is done [15].

The purpose of this paper is to conduct an empirical analysis of the determinants of participation in mammography screening. The analysis focuses on European women aged 50-69 years. The data base used is the first and second wave of the Survey of Health Aging and Retirement in Europe (SHARE). The impact of physician quality on screening decisions will be of special interest. No empirical study so far includes physician quality as a potential factor for the decision for screening. The reason is that survey data including this information are scarce. ^C ^Physician quality can be expected to influence the decision for screening, since asymmetric information is particularly widespread in health care markets often forcing expert physicians to act on behalf of their less informed patients [16]. Furthermore, individual perception of risks is often biased (e.g. [17]). Breast cancer is no exception in this regard and even women with a high risk of getting breast cancer tend to have false perceptions of the risks and the seriousness of breast cancer [18]. For this reason, physicians often need to act as agents for their less-informed patients, and they play an important role in determining mammography screening take-up. Empirical evidence clearly indicates that women follow physician advice for mammography screening (e.g. [19,20]). Thus, we hypothesize that a better physician - as measured by an index defined in the next section - will more often suggest mammography screening in line with the official national and EU screening guidelines, thus inducing higher screening rates.

A second focus lies on the impact of subjective live expectancy on mammography screening. Economic theory suggests [21,22] that the motivation to invest in one's own health should depend on the subjective life expectancy. Women who expect a longer life should be more inclined to invest in health in order to spend more years in good health than women expecting to live only for another few years. A corresponding phenomenon has been empirically detected for smoking behaviour in the US-context by Fang et al. [23] who call it the "Mickey Mantle Effect".^D ^However, empirical analysis has to consider that stated life expectancy suffers from measurement error, leading to attenuation bias. Moreover life expectancy may be endogenous mainly due to reverse causality: investment in health increases life expectancy. We follow Fang et al. [23] and apply their empirical approach to mammography take-up and control for measurement error and endogeneity of the subjective life expectancy through an IV-approach.

The results show that better physician quality, better education, being married, being a "high user" of health care, younger age and better health are associated with higher rates of screening take-up. Moreover subjective life expectancy strongly influences screening probability once the measurement error is controlled for.

The remainder of the paper is organized as follows: The next section provides some information on the data set and the theoretical and empirical screening determinants. Then the empirical strategy is described, followed by the results. The final section discusses the results and adds some concluding remarks.

## Data, determinants of mammography screening and variables

### Data

We use data from the first (2004) and second (2006) wave of the Survey of Health, Ageing and Retirement in Europe (SHARE) to analyze the determinants of mammography screening. SHARE is a large representative micro data set of more than 30,000 individuals above the age of 50 years from 14 European countries and Israel starting in 2004. It provides detailed information on health status and on a variety of other socioeconomic characteristics. The data was collected using a computer assisted personal interviewing (CAPI) program, supplemented by a self-completion paper and pencil questionnaire.^E ^This "drop-off questionnaire" includes additional questions which address issues like physician behaviour or utilization of preventive health care. Furthermore it includes a question about mammography take-up in the last two years. This questionnaire was only sent to a subgroup of the sample and no respondent received it in both waves.

We restrict our sample to women aged 50-69 years, since for this group mammography screening is officially recommended at both European level and the national level of the countries included. In addition, we exclude women if they reported a history of cancer as they are not representative and we discard observations with missing or unreliable values for the variables of interest and the other explanatory variables. Therefore, our estimation sample consists of two cross-sections with 6,893 women in total (4,412 from the first wave surveyed in 2004 and 2,481 from the second wave surveyed in 2006).

Table 1 provides descriptive statistics of the variables included in the empirical analysis.

**Table 1:** Sample Means and Description Variable Definition

Variable	Definition	Mean (N = 6,893)
*Variables from Wave 1 and 2 of the SHARE Dependent Variable*		
Mammogram	Mammogram screening in the last two years (Yes = 1, No = 0)	0.647
*Explanatory Variables*		
Physician Quality Index	GP quality between 0 and 1 as explained in the text	0.301
Life Expectancy	Self-stated probability of being alive in about 10 years	0.670
50 ≤ Age < 55		0.288
55 ≤ Age < 60		0.273
60 ≤ Age < 65		0.243
65 ≤ Age < 70		0.195
Self Assessed Health	Excellent = 1 to poor = 5	2.907
Limitations in ADL	Number of limitations in Activities of Daily Living	0.113
Heart Attack	Chronic Conditions: Heart attack	0.067
Stroke	Chronic Conditions: Stroke	0.019
Diabetes	Chronic Conditions: Diabetes	0.074
Lung disease	Chronic Conditions: Lung Disease	0.041
Has Partner	Binary Variable for whether to women has a partner	0.744
Children in HH	Number of children living in household	0.462
ISCED Low	Education ISCED^1 ^level between 0 and 2	0.444
Doctor visits ≥ 10	Number of doctor visits ≥ 10 within the previous 12 month	0.221
No drugs	Binary Variable for whether the woman regularly takes prescription drugs	0.314
Hospital Stays ≥ 2	Number of hospital stays ≥ 2 within the previous 12 month	
*Year and Country Dummies*		
Year 2006		0.361
Austria		0.079
Germany		0.098
Sweden		0.068
Netherlands		0.088
Spain		0.068
Italy		0.093
France		0.071
Greece		0.044
Switzerland		0.059
Belgium		0.099
Czech		0.068
Poland		0.068
Ireland		0.038
Denmark		0.059
*Instruments*		
Father Age at		0.262
Death ≤ 65		
Father Age at Death		0.111
65 to 69		
Father Age at Death		0.111
70 to 74		
Father Age at Death		0.126
75 to 79		
Father Age at Death		0.139
80 to 84		
Father Age at		0.093
Death ≥ 85		
Age Father_IV	Age at death or current age of father if still alive	72.02
Mother Age at		0.144
Death ≤ 65		
Mother Age at Death		0.070
65 to 69		
Mother Age at Death		0.092
70 to 74		
Mother Age at Death		0.116
75 to 79		
Mother Age at Death		0.125
80 to 84		
Mother Age at		0.118
Death ≥ 85		
Age Mother_IV	Age at death or current age of mother if still alive	76.64

^1^International Standard Classification of Education

### Determinants of mammography screening and variables

Economic theory suggests all variables mentioned in Table 1 could be important determinants of individual screening decision. From an economic perspective the decision to undergo mammography screening is an investment decision. Such an investment is worthwhile if the expected present value of the reduction in disease and in the probability of death is larger than the opportunity costs of the intervention (comp. [8,9,24,25] for a formalization of these notions). However, the question whether people actually decide to invest in mammography screening is largely an empirical one as we will argue in the following paragraphs. The paragraphs discuss certain hypotheses that would seem to be implied by economic theory and relate them to existing empirical results. Moreover the paragraphs describe the variables that we use in the empirical analysis in order to test the hypotheses.

### Age

First of all, according to economic theory, age should influence mammography screening decision but the theoretical impact is offsetting. According to health human capital models (based on [8]) health depreciates at an increasing rate as one gets older, reducing the returns on investment. Moreover, the potential years of life saved due to mammography screening decline with age [9]. Alternatively, older women should be more likely to take-up mammography screening, because they have a greater risk for breast cancer than younger women (e.g. [26,27]) and thus expected benefits to mammography screening should be higher for those at higher risk for breast cancer. The great majority of empirical studies however indicate that older women are less likely to engage in mammography screening (e.g. [28,29]). To account for age we include dummy variables of different age groups. The reference age group that is not included in the regression is the group of women aged 50-54 years.

### Health status

Health Status should also be associated with the decision for screening. Those in poorer health should be more likely to undergo mammography screening, since they potentially have higher cost to getting other diseases. For example rehabilitation and treatment may be more difficult for people in poor health than for those who are otherwise in good health [30]. Alternatively, it may be the case that people in poor health have less time to receive treatment or screens given their physical limitations. Furthermore women - as well as the physician acting as their agent [16]^F ^- could set priority on other medical measures when sick, since mammography is associated with a future related and uncertain benefit [31]. Overall, it remains largely an empirical question whether poor health is associated with more or less mammography screening. The empirical literature is inconclusive whether poor health is a barrier to screening. To address this question, we control for health using a detailed set of health indicators. These include self-assessed health (i.e. excellent, very good, good, fair, or poor), as well as a number of objective measures such as binary indicators for whether the respondent was ever diagnosed with stroke, heart disease, lung disease and diabetes as well as an index of limitations in activities of daily living (ADLs).^G ^ADLs refer to daily self-care activities within an individual's place of residence, in outdoor environments, or both.

### Education and cognitive abilities

Better education may increase the use of screening services, implying more efficiency in producing health (e.g. [8]). For example, a better educated woman may be more likely to understand the benefits of mammography screening. In addition, these women may be more prone to recognize the early warning signs of breast cancer and be more apt to visit a physician when symptoms first occur. Education is captured by a dummy variable for low education as defined by ISCED equivalents.^H ^Since educational attainment in the past might not fully mirror the current skills to process information [32], we also analyse the role of current cognitive abilities. An increasing body of research suggests that differences in cognition partly explain variations in health behaviours (compare [33]). Cutler and Lleras-Muney [34] show that disparities in cognitive ability can account for about 30% of the average education gradient in a wide variety of health behaviors in Great Britain and the United States. In addition, cognitive ability at later ages seems to be more important than that measured earlier in life [33]. In this paper cognitive abilities are captured by the variables "recall" and "verbal fluency". Both are often used as proxies for cognitive abilities in empirical work (e.g. [35]). Verbal fluency is measured by the number of different animals the respondent is able to state within one minute. Recall is measured by the number of words the respondent can recall from a list of ten words that has been shown her some minutes before. Both measures reflect cognitive functions as identified by the cognitive psychology literature [27]. Empirical studies (e.g. [36]) find that cognitive impairment is associated with lower screening mammography rates. We hypothesize that the worse the cognitive skills the lower the probability to screen, since cognitive impairments limit the patients' ability to gather and process information.

By increasing the actual or perceived costs of processing information, they can act as a barrier for mammography screening. The positive influence of information on the demand of prevention is shown by Parente et al. [37], who find that consumer knowledge has a substantial positive effect on the use of preventive services.

### Family structure

Having a partner should be associated with higher screening rates as empirical studies reveal [34,38]. Responsibility for the partner might be important, since spouses seem to encourage each other to a health-promoting behaviour [39]. Those who have a partner and/or have children are probably reminded more often of the importance of mammography by their loved ones. Thus living not alone should lower information costs. We include controls for the number of children living in the household and a dichotomous variable for whether a woman has partner.

### High users

Another factor for the screening decision should be how much prior health care one has used. Some individuals are simply "high users" of medical care, while others may choose not to utilize health care, even when it is readily available and affordable. Wu [28] finds empirical evidence for this regarding mammography screening in the US-context. To address "using behaviour" in the European context, we follow the empirical strategy of Wu [28] and include three dichotomous variables for whether a women i) had at least ten doctor's office visits in the last year, ii) more than two hospital stays in the last year and iii) regularly takes prescription drugs.

### Life expectancy

The motivation to invest in one's own health should depend on the subjective life expectancy of the respondent as well. Individuals who expect a longer life should be more inclined to invest in health, since the potential payoff of health investments is greater for people in good health than for people who believe to live for a few more years only [21]. Fang et al. [23] find that individuals who expect a longer life are significantly less likely to be currently smoking. They find no effect, however, for other health behaviours like heavy drinking or obesity. We calculate a variable indicating subjective life expectancy from following question of the SHARE: What are the chances that you will live to be age 75/80/85/90/95/100/105/110/120 or more?" (either 75 or current age plus about 10 years). Based on panel data from the Health and Retirement Study, Hurd and McGarry [40] analyzed the ability of subjective survival probabilities to predict actual mortality. Using panel data they found that subjective survival probabilities are an adequate measure to predict actual survival: those who survived in the panel reported survival probabilities approximately 50% higher at baseline compared to those who died.

### Physician quality

Finally, we want to analyse the impact of physician quality on the decision to undergo mammography screening. As argued by Maurer [41], health literacy of the typical patient is limited, and patients rely heavily on their physician's advice. Usually, they follow their doctor's recommendation, which also applies to mammography screening. May et al. [19] find in an US-study that 66% of women who received a recommendation adhered and of women receiving a documented recommendation, 75% adhered. Alternatively Meissner et al. [20] found for the US that 80% of non-screeners who reported having access to health care did not receive a recommendation for a mammogram.

Unfortunately, we cannot test directly for the impact of physician's advice on the probability of screening decision. Therefore we follow Maurer [41] as well as Schmitz and Wübker [42] and compute a physician quality score and assume that better physicians are more likely to recommend mammography screening.^I ^The idea behind this assumption is that better physicians are more prone to follow national guidelines regarding mammography screening and thus are more likely recommend screening for women if it is indicated (i.e. women aged 50 to 69 years). The quality score is computed in the following way. We use the answers of individuals to five questions in the drop-off questionnaire concerning specific geriatric assessments, which any general practitioner should routinely perform. These are how frequently a doctor i) asks about physical exercise, ii) suggests regular physical exercise, iii) asks about falls, iv) checks balance, and v) asks about drugs used. We sum up all the answers where we assign the category "at every visit" a 2, "at some visits" a 1 and "never" a 0. Like Maurer [41] and Schmitz and Wübker [42] we acknowledge that some questions are the more important the older the women are and the less important the younger the women are. Therefore, we do not consider balance checks and queries about falls if the respondent is aged 50-59. For women aged 60-69 we weight the answers to these two questions with 0.5. To get a quality indicator that falls into the range of 0 and 1 we divide the sum by the age-adjusted maximum possible number of points. Clearly not performing these geriatric assessments at every visit does not automatically reveal a bad physician quality. The lack of time in the daily routine might cause that a doctor does not perform these aspects regularly and this may not automatically reflect that the doctor does not assign women to mammography if it is indicated. However, we think it is reasonable to assume that physicians who perform these assessments regularly are on average more prone to recommend a mammography screening if it is indicated compared to those who do not perform these aspects regularly. This is supported by a strong positive correlation between the quality-score and the recommendation of a colonoscopy by a physician if it is indicated which is also investigated in the SHARE.^J^

## Empirical strategy and estimation results

### Basic analysis

We apply two basic regression models: First the linear probability model, that is, an OLS-regression of the variable indicating a mammography screening on the above-mentioned exogenous variables.^K ^Second the probit regression model, which in contrast to the OLS-regression imposes the restriction that a predicted value lies inside the range of 0 and 1. To control for institutional and cultural differences in screening behaviour we include a full set of country dummies. Table 2 reports the results of the linear probability model in the first column.

**Table 2:** Estimation Results from OLS and Probit

	(OLS) Mammogram		(Probit) Mammogram	
Physician Quality Index	0.107**	(0.041)	0.116***	(0.042)
Life Expectancy	0.065**	(0.030)	0.072**	(0.031)
55 < = Age < 60 (d)	0.015	(0.015)	0.020	(0.018)
60 < = Age < 65 (d)	0.005	(0.013)	0.007	(0.015)
65 < = Age < 70 (d)	-0.095**	(0.032)	-0.102***	(0.035)
Self Assessed Health	-0.009**	(0.004)	-0.011**	(0.005)
Number of ADL	-0.029**	(0.011)	-0.032***	(0.012)
Heart Attack (d)	-0.020	(0.021)	-0.023	(0.024)
Stroke (d)	-0.083**	(0.031)	-0.099***	(0.036)
Diabetes (d)	-0.055**	(0.019)	-0.063***	(0.021)
Lung Disease (d)	0.012	(0.025)	0.010	(0.030)
ISCED Low (d)	-0.056**	(0.020)	-0.066***	(0.021)
Verbal Fluency	0.002*	(0.001)	0.003**	(0.001)
Recall Delayed	0.001	(0.004)	0.000	(0.004)
Has Partner (d)	0.046***	(0.014)	0.053***	(0.016)
Children in Household	-0.008	(0.013)	-0.009	(0.014)
Doctor Visits = 10 (d)	0.041**	(0.016)	0.046***	(0.018)
Regularly Drugs (d)	0.055***	(0.010)	0.064***	(0.011)
Hospital Stays = 2 (d)	0.019	(0.047)	0.016	(0.051)
*Year and Country Dummies*				
Year 2006	0.006	(0.010)	0.007	(0.013)
Austria (d)	0.477***	(0.009)	0.318***	(0.004)
Germany (d)	0.251***	(0.006)	0.209***	(0.004)
Sweden (d)	0.634***	(0.008)	0.365***	(0.001)
Netherlands (d)	0.645***	(0.011)	0.378***	(0.002)
Spain (d)	0.470***	(0.021)	0.312***	(0.007)
Italy (d)	0.452***	(0.018)	0.314***	(0.008)
France (d)	0.614***	(0.010)	0.362***	(0.002)
Greece (d)	0.225***	(0.020)	0.191***	(0.014)
Switzerland (d)	0.271***	(0.010)	0.217***	(0.007)
Belgium (d)	0.538***	(0.009)	0.349***	(0.003)
Czech (d)	0.343***	(0.021)	0.257***	(0.012)
Poland (d)	0.238***	(0.025)	0.200***	(0.018)
Ireland (d)	0.285***	(0.023)	0.224***	(0.015)

Observations	6,893		6,893	

Marginal effects; Standard errors in parentheses; (d) for discrete change of dummy variable from 0 to 1

* *p *< 0.10, ** *p *< 0.05, *** *p *< 0.01

The results indicate that physician quality has a positive and significant impact on the decision to undergo mammography screening. ^L ^Specifically, our estimates show on average 10.4%age points higher screening rates among women whose family physician performs all geriatric assessments relative to those whose doctor does not undertake any evaluation. ^M^

Given that the average screening rate in this age-group is 64 percent, this is a considerable amount.

Besides physician quality, the main variable explaining the demand for mammography screening is age. Older women are less likely to get mammograms. In example, being in the 65-69 age group decreases the probability of getting a mammography screening by 9.5 percentages points compared with the 50-54 age group (i.e. the reference group that is not included in the regression model). Generally, sicker women as measured by objective and subjective measures of health status are less likely to get mammograms. More precisely, lower ability to perform activities of daily living and suffering from chronic conditions like a stroke and diabetes are similarly associated with lower screening rates. Moreover, even after controlling for those objective measures of health status, worse self assessed health is associated with lower mammography screening rates. Education and the capability of processing information affect the mammography screening decision. Both having a higher than low education and yielding more points in the verbal fluency test are associated with higher screening rates. The number of children in the household does not seem to play an important role, whereas whether a woman has a partner significantly increases the screening probability.

Being "high-users" of medical care is significantly positive associated with mammography screening. Specifically, our estimates show on average 5.5 percentage points higher screening rates among women who regularly take drugs compared to women who do not take drugs regularly. Moreover, women who had at least ten doctor visits in the last year have on average 4 percentage points higher screening rates than those with less than ten doctor visits.

Finally, higher subjective life expectancy significantly increases the likelihood of getting a mammogram. A 10%age point increase in subjective life expectancy reduces probability of mammography screening by about 0.6 percentage points. The results for the probit estimation are quite similar to those of the OLS estimation for most variables as can be seen in the right column of Table 2. Noteworthy is, however, that the coefficient of physician quality and life expectancy are a little bit higher using probit specification compared to OLS. Moreover, we find significantly smaller differences in the coefficients of the country dummies in the probit estimates, since the probit model imposes the restriction that a predicted value has to lie inside the range of 0 and 1.

### Measurement error and endogeneity concerns

**Figure 1:**
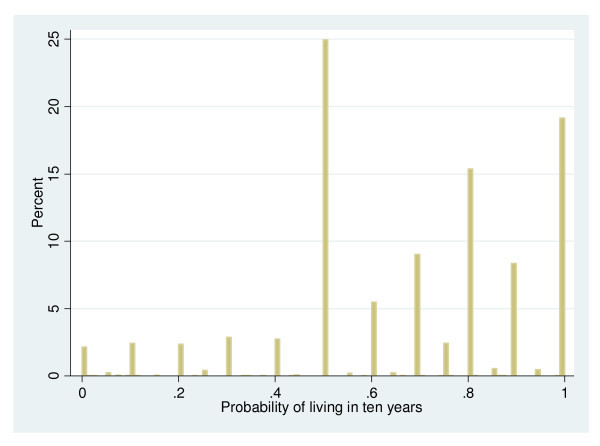
**Heterogeneity of survival beliefs and measurement error**.

As stated in the introduction, we are seriously concerned that subjective life expectancy as one of our most important covariates suffers from measurement error leading to attenuation bias. Measurement error arises if an explanatory variable is measured with additive random errors. The higher the part of variability that is due to errors, the larger is the magnitude of the attenuation bias (e.g. [43]). Hurd, McFadden and Gan [44] reveal that due to cognitive disability a lot of respondents systematically provided focal-point answers (0, 0.5 or 1) to the questions on subjective survival probabilities in the sample of older individuals (aged 70 and over) in the Study of Assets and Health Dynamics among the Oldest Old (AHEAD). A similar response pattern comes up for women aged 50-69 years in the SHARE-data with many focal answers at 0, 0.5 and 1 as shown by Figure 1. These cannot represent the true probabilities, both because the distribution of true probabilities should be continuous and because the true probabilities cannot be exactly either zero or one [44]. In the consequence the coefficient on that variable in an ordinary least squares regression will be biased towards zero.

In addition, following the discussion of Fang et al. [23] the impact of subjective life expectancy on investment in health is endogenous, mainly due to reversed causality. While investment in health might depend on subjective life expectancy (which is to be analysed here), subjective life expectancy is also likely to depend on investment in health. Individuals who generally invest more in health might believe in a pay-off of their behaviour, resulting in the expectancy of a longer life. We follow Fang et al. [23] and Bloom et al. [45] in using age at death of respondents' parents (or their current age if still alive), as well as age^2^, age^3 ^and binary indicators of whether the father or mother died at an age that fell in the range of under 65, 66 to 70, 71 to 75, 76 to 80, 81 to 85, or 86 and over.^N ^The instruments can be seen as proxies for health endowment like genetic factors that are transfused from parents to children. Individuals with older parents are likely to have a better health endowment than those whose parents died early, possibly due to a genetic disease.

The identifying assumption here is that genetic factors affect subjective life expectancy but not the decision to get a mammography screening once health and subjective life expectancy are controlled for. According to our strategy in the "Basic-Analysis" section, we perform both linear Instrumental Variable Two-Stage Least Squares (IV-2SLS) estimation (following Fang et al. [23]), and a non-linear two-stage procedure following Newey [50} to correct standard errors in the presence of a dichotomous dependent variable in the second stage. The 2SLS method is usually chosen even in cases where the dependent variable is dichotomous (e.g. Wooldridge [46]) since strong specification assumptions are required to justify the Newey [47] method. We present both for completeness and find quite similar results with all specifications.

Table 3 reports the results of linear and non-linear two-stage procedure.^O ^While the other coefficients remain quite stable when the instrumental variables regression is performed, the impact of subjective life expectancy increases.^P^

**Table 3:** Estimation results from IV OLS and IV Probit model

	(IV-OLS) Mammogram		(IV-Probit Model) Mammogram	
Second Stage Regression				
Physician Quality Index	0.108***	(0.039)	0.115***	(0.042)
Life Expectancy	0.256*	(0.131)	0.361*	(0.203)
55 < = Age < 60 (d)	0.012	(0.015)	0.015	(0.018)
60 < = Age < 65 (d)	-0.001	(0.012)	-0.003	(0.015)
65 < = Age < 70 (d)	-0.091***	(0.031)	-0.094***	(0.036)
Self Assessed Health	0.000	(0.009)	0.004	(0.012)
Number of ADL	-0.025**	(0.011)	-0.025**	(0.012)
Heart Attack (d)	-0.012	(0.019)	-0.009	(0.023)
Stroke (d)	-0.083**	(0.033)	-0.098**	(0.041)
Diabetes (d)	-0.048***	(0.018)	-0.051**	(0.022)
Lung Disease (d)	0.022	(0.027)	0.025	(0.031)
ISCED Low (d)	-0.054***	(0.020)	-0.061***	(0.021)
Verbal Fluency	0.002*	(0.001)	0.002	(0.001)
Recall Delayed	0.000	(0.004)	-0.001	(0.004)
Has Partner (d)	0.046***	(0.014)	0.052***	(0.017)
Children in Household	-0.007	(0.012)	-0.008	(0.014)
Doctor visits = 10 (d)	0.040**	(0.016)	0.045**	(0.018)
Regularly Drugs (d)	0.057***	(0.010)	0.065***	(0.011)
Hospital Stays = 2 (d)	0.022	(0.048)	0.020	(0.055)
*Year and Country Dummies*				
Year 2006	0.003	(0.009)	0.003	(0.013)
Austria (d)	0.493***	(0.016)	0.326***	(0.006)
Germany (d)	0.263***	(0.011)	0.220***	(0.007)
Sweden (d)	0.644***	(0.011)	0.368***	(0.003)
Netherlands (d)	0.649***	(0.010)	0.379***	(0.002)
Spain (d)	0.466***	(0.019)	0.309***	(0.008)
Italy (d)	0.453***	(0.017)	0.313***	(0.008)
France (d)	0.622***	(0.013)	0.365***	(0.003)
Greece (d)	0.236***	(0.024)	0.200***	(0.016)
Switzerland (d)	0.277***	(0.011)	0.221***	(0.007)
Belgium (d)	0.554***	(0.016)	0.356***	(0.006)
Czech (d)	0.387***	(0.036)	0.287***	(0.022)
Poland (d)	0.263***	(0.031)	0.223***	(0.022)
Ireland (d)	0.296***	(0.023)	0.231***	(0.014)

Observations	6,893		6,893	
F-Stat. instruments first stage	14.54			
Overid. Statistics^2^	27.15 (p = 0.101)			

Marginal effects; Standard errors in parentheses

(d) for discrete change of dummy variable from 0 to 1

* *p *< 0.10, ** *p *< 0.05, *** *p *< 0.01; ^2^p-values in parenthesis

The change is as predicted. It indicates that the effect of life expectancy is strongly underestimated when measurement error and endogeneity is not taken into account. Subjective life expectation heavily increases the probability of investing in one's own health (by taking a mammogram). Specifically, the estimates imply that a 10%age point increase in this subjective probability reduces probability of mammography screening by about 2.3 percentage points. This effect is much stronger than before (0.6 percentage points) and still significant on the 10% level. Possibly, this effect is too high, since the large standard errors associated with the IV approach leads to large 90% confidence intervals of [0.0073438; 0.4492358]. However, this result does not seem to be due to weak instruments as the F-statistics exceeds the Staiger-Stock rule-of-thumb of 10 [48], when testing for the exclusion of the instruments in the first-stage regression. Furthermore, over-identification tests support the validity of our instruments. If one is willing to assume that the mother's age of death is exogenous to the individual mammography screening decision, the hypothesis that all other instruments are valid cannot be rejected.

Again, most of the coefficients in the IV-Probit Model are quite similar to those of the IVOLS Model, as can be seen in the right column of Table 3. However, the impact of life expectancy is about 40 percent higher (0.361 versus 0.256) in the IV-Probit Model compared to the IV-OLS Model. Moreover, once more the coefficients of the country dummies in the IV-Probit estimates are significantly smaller compared to the IV-OLS estimates. However in both models they remain always jointly significant indicating huge differences across countries that are not picked up by individual differences in our observed variables ^Q^.

## Discussion and conclusion

Breast cancer is the main cause of cancer-mortality among women in Europe. Screening mammography helps to detect breast cancer before it becomes invasive, and mortality can be significantly reduced by regularly mammography screening. Moreover, for women aged 50- 69 years mammography screening has proven to be highly cost-effective. Even though mammography is officially recommended both on the national and European level for this group of women, screening rates in most European countries remain far from 100 percent and reasons for underparticipation remain unclear. The purpose of this paper was to conduct an empirical test of certain hypotheses implied by economic theory concerning the determinants of mammography screening focusing on European women aged 50-69 years using the SHARE data-base. The results indicate that better education, being married, younger age and better health consistently associated with higher rates of screening take-up. These results suggest that additional efforts may be needed to inform and convince the women living alone but also the elderly and women in poor health of the preventive benefits of mammography. Certain interventions such as invitations appointments and telephone calls have shown to be effective at increasing uptake (compare [13]).

The impact of physician quality and subjective life expectancy was of special interest in this paper. Having a family physician who generally complies with indicated geriatric assessments - as a proxy for physician quality - has a strongly significant positive effect on mammography screening propensity. Specifically, our estimates indicate on average 10.7 percentage points higher screening rates among respondents whose family physician performs all geriatric assessments relative to those whose doctor does not undertake any evaluation. This result is consistent with agency theory suggesting that physicians act on behalf of their less informed patients. This result may imply that interventions could address the physicians as key communicator since the percentage of physicians who recommend mammography screening in Europe may be too low. In example, a study from Switzerland reveals (compare [49]) that among clinically practising physicians, only 22% reported generally prescribing biannual screening mammography's for women aged 50-69. Thus, there might be a need to educate physicians regarding the preventive benefits of mammography screening.

Finally, we find that subjective life expectancy strongly influences screening probability once the measurement error is controlled for. Women who expect a longer life are much more inclined to invest in mammography screening than women who believe to live a few more years only. In terms of health policy implications, this result suggests that health promotions programs which include discussions that increase subjective survival expectations may provide suitable leverage to increase screening rates.

## End notes

^A ^The incidence rates are generally slightly increasing with age. For example in Denmark 319 per 100,000 women aged 50 to 69 years compared to 358 women aged 70+ got the diagnosis of breast cancer in 2002. But this pattern does not hold for each country [50].

^B ^There is serious controversy regarding the effectiveness and cost-effectiveness of mammography screening especially for women younger than 50 years and older than 70 years. Thus we focus on women aged 50-69 years.

^C ^Maurer [41] as well as Schmitz and Wübker [42] used physician quality to explain influenza vaccination decision in Germany and Europe using the SHARE. We base our paper on the quality score introduced by Maurer [41].

^D ^The phenomenon is named after the legendary American baseball player Mickey Mantle who exhibited a very unhealthy behaviour because he expected to die at an early age because several of his family members died of a rare hereditary disease at a young age.

^E ^For more details on the sampling procedure, questionnaire contents and fieldwork methodology, readers should refer to Börsch-Supan and Jürges [51].

^F ^For example Yaskaskas et al. [52] show that women with disabilities are less likely than those without disabilities to receive a physician recommendation for screening mammography.

^G ^This variable describes the number of limitations with activities of daily living (ADL). Six activities are included: Dressing, including putting on shoes and socks, Walking across a room, Bathing or showering, Eating, such as cutting up your food, Getting in and out of bed, using the toilet, including getting up or down.

^H ^The International Standard Classification of Education (ISCED) was designed by UNESCO in the early 1970's to serve as a tool to facilitate comparisons of education statistics and indicators of different countries on the basis of uniform and internationally agreed definitions. The higher the ISCED value the higher the education-level. The levels are as follows defined: Level 0: Pre-primary education; Level 1: Primary education or first stage of basic education; Level 2: Lower secondary or second stage of basic education; Level 3: (Upper) secondary education; Level 4: Post-secondary non-tertiary education; Level 5: First stage of tertiary education (not leading directly to an advanced research qualification); Level 6: Second stage of tertiary education (leading to an advanced research qualification, e.g. a Ph.D.). We define low education by ISCED values between 0 and 2.

^I ^The indicator used by Schmitz and Wübker [42] differs slightly from the one that Maurer [41] uses. The reason is, firstly, that one question ("How often does your GP check your weight?") is only asked in the first wave. Schmitz and Wübker [42] ignore this one and use only five instead of six questions. Furthermore, Maurer does not weight the answers but assigns a 1 if the GP asked a specific question during at least some visits. The pros and cons of this approach can be debated. On the one hand, Maurer's method comes with an information loss. On the other hand, his approach might be more robust to recall error as it is easier to remember if a GP ever asked this question than how regular she does. However, we tried both quality scores and did not find qualitative differences in our results. In addition we tested analogue to Schmitz and Wübker [42] different weighting schemes of the questions in order to test robustness of the results. We find only small differences in our results. The results are available upon request.

^J ^The data are available upon request from the authors. Most guidelines recommend an endoscopic examination (colonoscopy) of the colon from the age of 50 for both men and women [53] with a frequency that varies in order to detect colon cancer in its earliest stages. In the SHARE people are asked whether a physician recommended a colonoscopy in the last two years.

^K ^It turns out that less than 1% (55 observations) of all observations have a predicted value outside the range of 0 and 1. We feel that this is a reasonably low figure.

^L ^This basic result holds independently of the refinement of the physician quality measure.

^M ^Alternatively, women who are treated by a physician with a one standard deviation higher quality score have roughly 3 percentage points' higher screening rates.

^N ^Good instruments should show a considerable explanatory power for subjective life expectancy but must not affect the decision to undergo a mammography screening once the remaining explanatory variables are controlled for.

^O ^Additional file 1: Table A 1 reports the first-stage results from two-stage estimation. The dependent variable is self-stated probability of being alive in about 10 years. Clearly, health status as measured by self assessed health is a very important determinant of life expectancy. Moreover, parents' ages at death have large and significant effects in the expected direction. For instance, having a father who died between 65 and 69 years reduces the subjective probability of being alive in about 10 years by 4.5 percentage points, *ceteris paribus *(compared to those women, whose father is still alive). The F-test for the joint significance of the parental age at death variables is 14.54 indicating that the instruments are not weak.

^P ^Note, however that the coefficients measuring health status (e.g. Self Assessed Health) change in the IV-OLS compared to OLS. This is due to the correlation of the health measures with the instruments.

^Q ^Unfortunately there are no tests for a) weak instrument and b) over-identification of instruments in cluster-robust IV-Probit models. However, the results of the F-test and over identification test in the linear model support the validity of our instruments and do not indicate that the robustness of the results suffer from weak instruments.

## Competing interests

The author declares that they have no competing interests.

## Supplementary Material

Additional file 1 **Table A1**. First-Stage IV Regression Results: Predicting Life Expectancy. Click here for file
