# Supplementary material for: Who gets a mammogram amongst European women aged 50-69 years?
Source: Health Econ Rev. 2012 Apr 5;2:6. doi: 10.1186/2191-1991-2-6 (PMC3402934; doi:10.1186/2191-1991-2-6)
Supplement: Additional file 1 — Table A1. First-Stage IV Regression Results: Predicting Life Expectancy. [file 2191-1991-2-6-S1.DOC]

**Additional file 1**

Table A1: First-Stage IV Regression Results: Predicting Life Expectancy

|  | **Dependent variable**: Life Expectancy  i.e. Self-stated probability of being alive in about 10 years | |
| --- | --- | --- |
| Physician Quality Index | -0.002 | (0.013) |
| 55 <= Age < 60 | 0.010 | (0.008) |
| 60 <= Age < 65 | 0.021^*^ | (0.010) |
| 65 <= Age < 70 | -0.031^***^ | (0.010) |
| Self Assessed Health | -0.051^***^ | (0.004) |
| Number of ADL | -0.020^**^ | (0.009) |
| Heart Attack | -0.041^***^ | (0.013) |
| Stroke | 0.002 | (0.026) |
| Diabetes | -0.030^***^ | (0.009) |
| Lung Disease | -0.052^***^ | (0.016) |
| ISCED Low | -0.009 | (0.009) |
| Verbal Fluency | 0.002^***^ | (0.001) |
| Recall Delayed | 0.003 | (0.002) |
| Has Partner | -0.000 | (0.008) |
| Children in Household | -0.006 | (0.004) |
| Doctor visits ≥ 10 | 0.004 | (0.008) |
| Regularly Drugs | -0.007 | (0.006) |
| Hospital Stays ≥ 2 | -0.013 | (0.017) |
| *Year and Country Dummies* | | |
| Year 2006 | 0.014^***^ | (0.004) |
| Austria | -0.084^***^ | (0.003) |
| Germany | -0.062^***^ | (0.003) |
| Sweden | -0.057^***^ | (0.004) |
| Netherlands | -0.023^***^ | (0.004) |
| Spain | 0.012^*^ | (0.006) |
| Italy | -0.006 | (0.006) |
| France | -0.047^***^ | (0.003) |
| Greece | -0.062^***^ | (0.006) |
| Switzerland | -0.039^***^ | (0.004) |
| Belgium | -0.083^***^ | (0.002) |
| Czech | -0.218^***^ | (0.007) |
| Poland | -0.133^***^ | (0.011) |
| Ireland | -0.056^***^ | (0.010) |
| *Instruments* | | |
| Father Age at Death ≤ 65^++^ | -0.029 | (0.018) |
| Father Age at Death 65 to 69 | -0.045^**^ | (0.015) |
| Father Age at Death 70 to 74 | 0.001 | (0.016) |
| Father Age at Death 75 to 79 | -0.007 | (0.015) |
| Father Age at Death 80 to 84 | 0.010 | (0.008) |
| Father Age at Death ≥ 85 | -0.000 | (0.015) |
| Mother Age at Death ≤ 65^++^ | -0.032 | (0.031) |
| Mother Age at Death 65 to 69 | -0.024 | (0.015) |
| Mother Age at Death 70 to 74 | -0.015 | (0.015) |
| Mother Age at Death 75 to 79 | 0.014 | (0.012) |
| Mother Age at Death 80 to 84 | -0.002 | (0.013) |
| Mother Age at Death ≥ 85 | -0.014 | (0.015) |
| Age Father_IV^+++^ | 0.005 | (0.009) |
| Age Father_IV² | -0.000 | (0.000) |
| Age Father_IV³ | 0.000 | (0.000) |
| Age Mother_IV^+++^ | -0.001 | (0.010) |
| Age Mother_IV² | -0.000 | (0.000) |
| Age Mother_IV³ | 0.000 | (0.000) |
| Constant | -26.179^***^ | (8.198) |
| Observations | 6,893 |  |
| R-squared | 0.161 |  |
| F-Test for excluded Instruments | 14.54 |  |

Standard errors in parentheses, clustered by countries. ^*^ *p* < 0.10, ^**^ *p* < 0.05, ^***^ *p* < 0.01

++ The omitted categories for the father and mother age at death dummies are mother still alive and father still alive.

+++ Age in the age polynomials is either current age or age at death.
